# ABA and Bud Dormancy in Perennials: Current Knowledge and Future Perspective

**DOI:** 10.3390/genes12101635

**Published:** 2021-10-18

**Authors:** Wenqiang Pan, Jiahui Liang, Juanjuan Sui, Jingru Li, Chang Liu, Yin Xin, Yanmin Zhang, Shaokun Wang, Yajie Zhao, Jie Zhang, Mingfang Yi, Sonia Gazzarrini, Jian Wu

**Affiliations:** 1 Beijing Key Laboratory of Development and Quality Control of Ornamental Crops, Department of Ornamental Horticulture and Landscape Architecture, China Agricultural University, Beijing 100193, China; pwq211@126.com (W.P.); jiahuiliang1230@163.com (J.L.); lijingru12345@163.com (J.L.); lllliuchang@126.com (C.L.); xy161354@163.com (Y.X.); 18336073226@163.com (Y.Z.); wangshaokuncau@163.com (S.W.); zhao224736@163.com (Y.Z.); zj5820212@163.com (J.Z.); ymfang@cau.edu.cn (M.Y.); 2 Biology and Food Engineering College, Fuyang Normal University, Fuyang 236037, China; suijuanjuan@163.com; 3 Biotechnology Institute, Fujian Academy of Agricultural Sciences, Fuzhou 350001, China; 4 Department of Biological Sciences, University of Toronto, Toronto, ON M1C 1A4, Canada; sonia.gazzarrini@utoronto.ca; 5 Department of Cell and Systems Biology, University of Toronto, Toronto, ON M5S 3G3, Canada

**Keywords:** ABA, perennials, bud dormancy, hormone, sucrose, epigenetics

## Abstract

Bud dormancy is an evolved trait that confers adaptation to harsh environments, and affects flower differentiation, crop yield and vegetative growth in perennials. ABA is a stress hormone and a major regulator of dormancy. Although the physiology of bud dormancy is complex, several advancements have been achieved in this field recently by using genetics, omics and bioinformatics methods. Here, we review the current knowledge on the role of ABA and environmental signals, as well as the interplay of other hormones and sucrose, in the regulation of this process. We also discuss emerging potential mechanisms in this physiological process, including epigenetic regulation.

## 1. Introduction

Dormancy is an evolved trait of perennial plants that allows vegetative buds to survive harsh environmental conditions, and it was classified into three categories by Lang (1987): ecodormancy, caused by limitations in environmental factors; endodormancy, where the inhibition resides in the dormant structure itself, and paradormancy, inhibited by distal organs. Abscisic acid (ABA) is recognized as an essential phytohormone in dormancy regulation, especially as a central hub in seed dormancy [1,2,3,4], but its regulatory mechanism in bud dormancy is not well understood.

### 1.1. ABA Metabolism and Signaling

ABA biosynthetic and catabolic pathways are well understood [5,6]. ABA is de novo synthesized from the precursor isopentenyl diphosphate (IPP), which is further converted into lycopene after four desaturation steps of carotenoid. Lycopene undergoes cyclization and hydroxylation to generate zeaxanthin. The first step of ABA biosynthesis is initiated in plastid with epoxidation of zeaxanthin to all-*trans*-xanthophylls zeaxanthin and violaxanthin, catalyzed by zeaxanthin epoxidase (ZEP) [7,8]. Violaxanthin is further isomerized into *cis*-violaxanthin, which is cleaved by 9-*cis*-epoxycarotenoid dioxygenase (NCED) to yield xanthoxin, the first C_15_ intermediate of ABA biosynthesis [9]. Xanthoxin is transferred from the plastid to the cytosol, where it is converted to abscisic aldehyde by short-chain alcohol dehydrogenase. Finally, abscisic aldehyde is oxidized by aldehyde oxidase 3 to form ABA [5,9]. The catabolic pathway of ABA is mainly established through hydroxylation reaction. ABA can be hydroxylated at the C-8′ position by ABA 8′-hydroxylase, which is encoded by the CYP707A gene family. 8′-hydroxy-ABA is unstable and enzymatically isomerizes to generate phaseic acid [10,11]. In addition, ABA homeostasis can also be altered by enzymes during intracellular and intertissue-mediated transport, such as cytosolic UDP- glucosyltransferases (GTs) or β-glucosidases (BGs) [12,13,14]. In addition, ABA could be transported among cells and organs in plants. The active transporters are ATP-binding cassette (ABCG) transporters, NRT1/PTR FAMILY (NITRATE TRANSPORTER 1/PEPTIDE TRANSPORTER FAMILY; NPF), multidrug and toxic compound extrusion (DETOXIFICATION 50; DTX50), and AWPM-19 (ABA-INDUCED WHEAT PLASMA MEMBRANE POLYPEPTIDE-19) family proteins [6].

Over the past decades, the core components and the regulation of ABA signaling have been characterized. In 2009, soluble receptor proteins (PYRABACTIN RESISTANCE/PYRABACTIN RESISTANCE 1-LIKE/REGULATORY COMPONENT OF ABA RECEPTOR (PYR/PYL/RCAR)) for ABA in Arabidopsis were identified [15,16]. ABA co-receptor, ABI1 and ABI2, are members of clade A Type 2C PP2Cs protein (PROTEIN PHOSPHATASE 2Cs) and act as negative regulators of ABA signaling. PP2Cs inactivate SnRK2s (SNF1-related protein kinase 2s) by dephosphorylating their kinase activation loop and repress ABA response in the absence of ABA [17,18]. In the presence of ABA, PYR/PYL/RCARs interact with PP2Cs in which the phosphatase activity of PP2Cs is inhibited, facilitating activation of SnRK2s. Subsequently, SnRK2s activate ABA-RESPONSIVE ELEMENT-BINDING FACTORS (ABFs or AREBs), which then initiate transcription at ABA-responsive elements (ABREs) in the promoter regions of their target genes [19].

### 1.2. Bud Dormancy

Perennial bud dormancy can be classified into two forms based on the position of dormant vegetative organs, i.e., woody bud dormancy (aboveground) and geophytes dormancy (underground; corms, tubers, bulbs and rhizomes, underground adventitious buds on the crown and lateral roots).

With respect to bud dormancy in woody plants, there is a wide range of genetic variation within and between species and responses. Here, we take the woody model plant, *Populus*, as a main example. In autumn, with short photoperiod and low temperature, growth cessation, bud set, and dormancy of the bud meristem occurs sequentially in buds of populus [20]. Among environmental factors, light plays the dominant role in dormancy of most woody plants [21]. Early woody bud dormancy involves a series of states: cessation of vegetative growth, formation of terminal buds, arrangement of abscission layers in leaves, development of cold resistance, establishment of winter rest (endodormancy), and leaf fall [22]. Initiation of bud dormancy can be stimulated by environmental factors like short photoperiod, low or high temperature, low nutrition and water deficit. During the stage of winter rest, there are many metabolic and developmental activities happening in the dormant buds including respiration, photosynthesis, slow cell division, enzyme synthesis, production of growth stimulators, and dissipation of growth inhibitors. In buds, callose disrupts the symplastic pathway in all vascular cells, including sieve elements, by blocking the plasmodesmata, resulting in decreased flow of water, nutrients and other molecules in buds [23]. Phytohormones including abscisic acid (ABA), gibberellin (GA), ethylene (ET), auxin and cytokinins (CKs) are involved in bud dormancy, of which ABA plays an essential role in this process [24]. DAM (DORMANCY-ASSOCIATED MADS-BOX) is a SHORT VEGETATVIE PHASE (SVP) homolog, which responds to environment factors including temperature and photoperiod, and regulates endogenous ABA levels during bud dormancy [25].

As for geophytes, the dormant organs develop in the soil before going to a dormant state. During bulb formation, endogenous ABA is increased, which stimulates starch accumulation and bulb development. Afterwards, increased ABA inhibits organs development and initiates bulb dormancy. Based on the different dormancy traits, the major geophytes can be classed into three groups: (1) species with a relative long dormant period, during which the differentiation of new organs is inhibited, like *Gladiolus*; (2) species that differentiate flower buds inside the bulbs before/during bulb dormancy, including *Tulipa*, *Asiatic Lilium* Hybrids and *Hyacinthus*; (3) species with no visible dormancy unless in hash environments, like *Hippeastrum*. Environmental factors (low temperature and short photoperiod) triggers bulb dormancy. Temperature is the main factor in geophytes dormancy, affecting internal metabolites, phytohormones and signal response [26,27]. During geophytes dormancy release, several metabolites change dramatically, including a gradually decreased ABA, increased GA and glucose/sucrose ratio and enhanced respiratory activity, although there are invisible morphological differences [28,29]. Other special chemicals like glycerol, phenolics and peroxidase in scales could regulate bulb dormancy as well [30,31].

Much progress has been achieved on seed dormancy in model plants like Arabidopsis, *Zea mays* and rice, although research on dormancy in perennial plant is also expanding. This article attempts to mainly review the existing knowledge of winter bud endodormancy in perennials including trees and geophytes. We restrict the review to broadleaf deciduous angiosperms living in seasonally cold environments and discuss the scientific questions that still need to be addressed in the near future.

## 2. ABA Integrates Environments Signaling in Regulating Bud Dormancy

### 2.1. ABA Mediates Photoperiod Response of the Bud Dormancy

In temperate ecosystems, the photoperiod is one of the most reliable indexes of the seasonal growth cycle. Light regulates plant dormancy through the circadian clock and flowering pathways [32,33]. In many tree species, the photoperiod (short days; SDs) is below the critical threshold for growth before the onset of winter-induced bud growth cessation [34]. Different light wavelengths are sensed by different photoreceptors, among which the phytochromes (phys) sense red (R) and far-red (FR) light [35]. phyA is the photoreceptor of FR light, while phyB is the R light photoreceptor as well as a thermosensor [35,36]. phyA and phyB transduce the light signals predominantly through their interactors (PHYTOCHROME-INTERACTING FACTORS; PIFs) [37]. In 2006, CO/FT (CONSTANS/FLOWERING LOCUS T) was first revealed to modulate light-regulated perennial bud dormancy in aspen trees. *FT* is induced by long days and downregulated by SD, and negatively regulates bud growth cassation by promoting the expression of *D-type cyclins* [38,39]. Other regulators in biological clock and flowering pathways are involved in bud dormancy as well, like MADS-box transcription factor FLOWERING LOCUS C (FLC) and SVP, SUPPRESSOR OF OVEREXPRESSION OF CONSTANS 1 (SOC1), LEAFY and so on [40,41,42].

ABA is induced by SD and disrupts intercellular communication in buds, promoting bud endodormancy. In this process, *SVP-LIKE* (*SVL*), an essential gene in bud dormancy, is induced by SD and positively regulates *NCED* expression, forming a positive feedback regulation with ABA signaling [33,43]. Moreover, SVL upregulates transcript level of CALS1, which encodes for a callose synthetase. CALS1 promotes callose deposition, leading to the closure of plasmodesmata with callosic plugs (dormancy sphincters) and limiting access to growth-active signals, thus blocking intercellular communication and slowing down cell activity in buds [33,41,44]. SVL also stimulates *BRC1* (*BRANCHED 1*) expression under SD and further inhibits *FT* expression, thus promoting bud dormancy [45]. BRC1 is negatively regulated by *LAP1* (orthologous to the Arabidopsis floral meristem identity gene *APETALA1*) under SD, forming a negative feedback loop that controls seasonal growth by interacting with and antagonizing FT [45]. In addition, ABA signaling genes such as *ABI3*, which is induced by SD-mediated increased ABA, is expressed in tissues including the young embryonic leaves, the subapical meristem, and the procambial strands, resulting in bud dormancy with incorrect bud formation [46]. ABI3 could physically interact with FDL1 (FD-Like 1) under SDs and regulate the expression of genes involved in bud maturation and adaptive responses for cold tolerance [47]. Recently, a chromodomain protein PICKLE (PKL) was shown to be inhibited by ABA under SD. In *abi1-1* plants, defects in dormancy regulation caused by ABA insensitivity can be suppressed by down-regulating *PKL* expression [33].

### 2.2. ABA Mediates Temperature Signals Regulated Bud Dormancy

Temperature is possibly the most important factor in regulating bud dormancy especially for geophytes whose dormant organs are embedded in the soil [48]. For many deciduous plants, e.g., *Malus pumila*, the growth cassation occurs in summer, with long photoperiod and high temperature, while the endodormancy happens in autumn, during SD and low temperature [49]. In most deciduous angiosperms, bud dormancy is followed by blockage of vessels with accumulated callose, which results in decreased metabolic function and transport between buds and branches [23]. In autumn, short-term cold exposure increases endogenous ABA content in perennial buds by CBF, which binds and activates *DAM/SVL* transcription, thus increasing ABA levels and inducing bud endodormancy [50,51]. ABA could further activate its downstream signaling factors, i.e., ABF2, ABF3 and HB22, which in turn regulate the expression of *DAM/SVP* during dormancy induction and release [50,52,53]. ABF2 interacts with *TCP20*, which directly inhibits *DAM* genes expression [52]. *DAM* could also be activated by the ICE (Inducers of CBF Expression)-CBF module under cold stress [24,51]. As mentioned above, phyB is also a thermosensor, which is a positive regulator of thermo-controlled bud break in poplar. phyB interacts with PIFs and inhibits its expression, leading to activation of *FT2* and repression of *BRC1* and *CENL1*, which promote bud break and growth [54].

After exposure to chilling temperatures, there is an increase in cell connectivity in buds when endodormancy is releasing [23]. Many studies have shown that long terms of cold treatment decrease ABA content by inhibiting ABA biosynthesis while activating ABA catabolism in dormant organs, including tree’s buds and geophytes, like poplar, pear, *Gladiolus hybridus* and *Leafy spurge* [21,29,34,53]. Prolonged chilling activates *CYP707A* genes expression, thus decreasing ABA content in dormant buds. Furthermore, DAM/SVP would be inhibited and followed by activation of genes related to cell cycle, cell expansion, GA biosynthesis and FT [50]. In poplar, low temperature upregulates *EBB1* (*EARLY BUD-BREAK1*), an APETALA 2 (AP2)-family transcription factor, resulting in suppression of *SVL* expression. Repressing *SVL* expression breaks the SVL/ABA feedforward loop, leading to the upregulation of *EBB3* and consequently to activation of *CYCD3.1*, and bud dormancy release [55,56].

**Figure 1:**
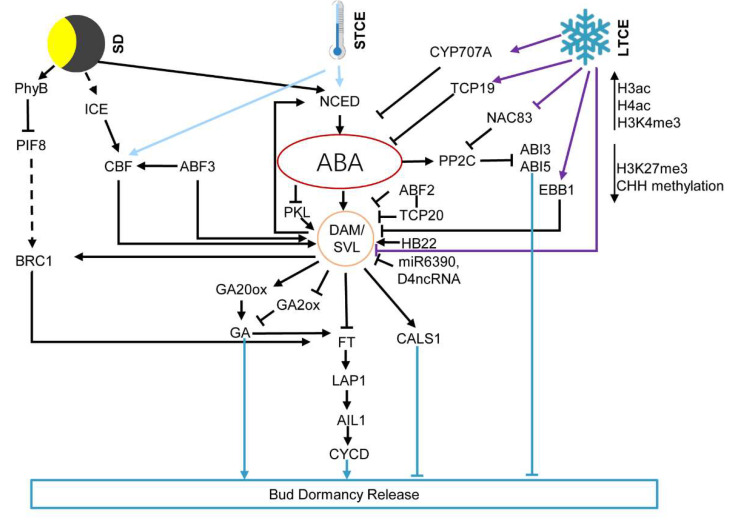
ABA integrates environments signaling in regulating bud dormancy. During dormancy induction, SD and STCE stimulate ABA biosynthesis and induce bud dormancy via DAM and ABA signaling that blocks the plasmodesmata and slow down the cell cycle. After long term of chilling, endogenous ABA is decreased by *cis*- and *trans*- regulation. DAM/SVL is repressed when ABA was decreased, resulting in promoted GA in cells and degradation of callose at the plasmodesmata. Enhanced cell communication leads to active cell division and bud break. Note that this overview summarizes interactions reported in different species, which are not necessarily occurring simultaneously. Full lines and dashed lines specify established and putative/indirect regulation, respectively. LTCE: long term cold exposure; SD: short day; STCE: short term cold exposure.

In addition to the DAM hub, there are several reports showing that ABA signaling mediates cold storage and controls bud dormancy release (Figure 1) [27,29]. In *Gladiolus*, the transcription level of *SVL* does not significantly change during corm dormancy release, but ABA dominantly regulates this process [27]. Cold storage inhibits *NAC83* expression in dormant corms and activates NAC83-targeted gene, *PP2C1*. *PP2C1* is the ABA co-receptor that plays roles in DNA duplication in dormant buds and inhibits ABA signal response, promoting corm dormancy release [27]. Cold storage could also activate *TCP19*, a Class I member of TCP family, which binds to the *NCED* promoter and represses *NCED* expression, contributing to reduction in endogenous ABA and corm dormancy release [57]. *TCP19* plays a role in cell division in buds as well, by positively regulating cyclin A/B/D genes during corm dormancy release [57].

## 3. Cross-Talk between ABA and Sugars in Bud Dormancy

Sugars provide energy to cell activity and also act as signaling molecules that regulate plant development and growth, including bud dormancy [58,59,60]. There is crosstalk between sugar signaling and other signaling pathways, including light and plant hormones (e.g., ABA and ethylene) [61,62,63]. Sugar signaling is essential for maintaining paradormacy by affecting cell cycle at the G1/S phase, and the transition from paradormancy to endodormancy in buds [64,65,66,67]. During bud dormancy release, endogenous starch content, which is high in accordance with endogenous ABA level, decreases [65,68]. Meanwhile, soluble sugars (glucose, sucrose, fructose, trehalose, etc.) increase during the transition from endodormancy to ecodormancy in buds of species such as leafy spurge, lily, sweet cherry and tree peony [26,65,69,70,71]. At the bud dormancy induction stage, sucrose in parenchyma cells declines and Tre6P (Trehalose 6-phosphate) decreases, which activates SnRK1 activity and stimulates ABA biosynthesis [72]. Generally, low Tre6P levels and/or high SnRK1 activities are associated with a dormant state/growth cassation, whereas high Tre6P levels and/or low SnRK1 activities are associated with active developmental progression [73]. For example, *TPP*-overexpressed (Tre6P-phosphatase) potato tubers accumulated higher levels of glucose and sucrose and sprouted prematurely, while tubers of *snrk1* mutant displayed strongly delayed sprouting [74].

ABA has been shown to inhibit sucrose transporters in vine and potato dormant buds [72,75]. Moreover, ABA promotes starch accumulation in the dormant phase in grape bud by increasing the expression of starch biosynthesis genes *SOLUBLE STARCH SYNTHASE 1* (*SS1*) and *SS3*, and inhibiting the expression of starch metabolism genes *INVERTASE*s (*INV*s) and sucrose biosynthesis genes *SUCROSE PHOSPHATE SYNTHASE*s (*SUP*s) [76].

**Figure 2:**
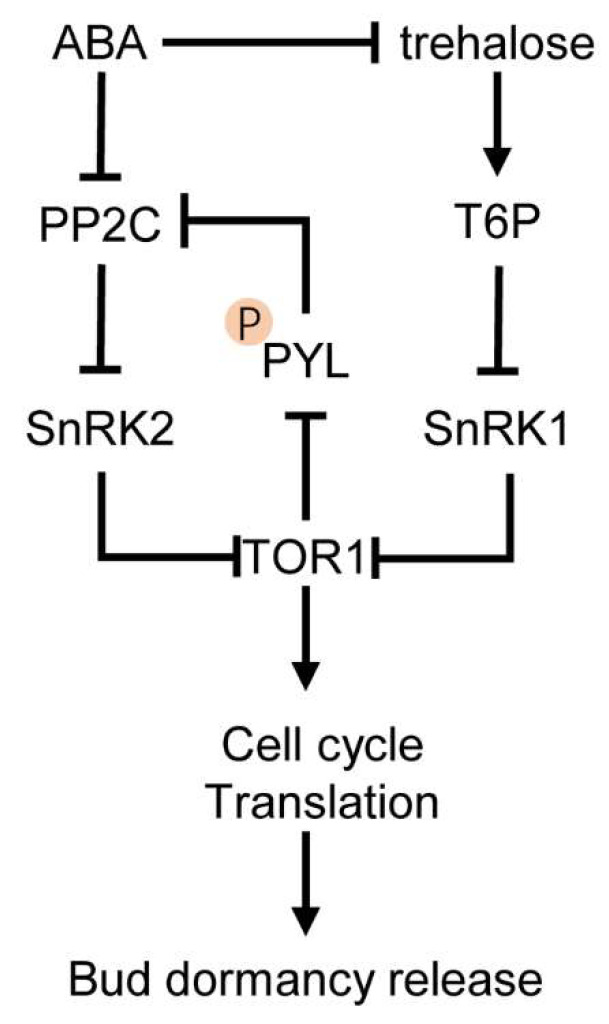
Schematic overview of the SnRK1-TOR-SnRK2 cascade in mediating the crosstalk between ABA with trehalose during bud dormancy release. SnRK1 signaling keeps and switches energy used for rapid growth and development toward enhanced stress tolerance and survival with low energy. SnRK1 can be functional by repressing plant growth and the activity of TOR kinase. In addition, SnRK1 signaling cross-talks with and actives ABA signaling together with SnRK2. SnRK2 represses TOR signaling by direct phosphorylation, leading to TOR complex dissociation. In favor conditions, TOR kinase backwards represses SnRK2 signaling via phosphorylation of the PYR1-LIKE (PYL) and active PP2C. TOR1 promotes the transcription of genes involved in cell-cycle progression and translation of ribosomal protein mRNAs in plants. SnRK1 is repressed by high energy signals, such as trehalose-6-P (T6P). Note that this overview summarizes interactions reported in different species or environmental condition, which are not necessarily occurring simultaneously. Full lines and dashed lines specify established.

In plants, the crosstalk between sugars and ABA can be mediated by the Tre6P-SnRK1 and SnRK1-TOR (Target of Rapamycin)-SnRK2 modules (Figure 2). TOR is a Ser/Thr protein kinase that belongs to the phosphatidylinositol 3-kinase-related lipid kinase family and stimulates cell cycle and mRNA translation by phosphorylating of the ribosomal protein S6 kinase S6K1 [77]. As mentioned above, low energy [e.g., low Tre6P, glucose-6-P (G6P), and glucose-1-P (G1P)] activates SnRK1 through ABA signaling [78]. Under stress conditions, SnRK1 and SnRK2 can repress TOR kinase by direct phosphorylation, resulting in TOR complex dissociation [79]. In the absence of ABA (or under low levels of ABA), PP2C phosphatases target SnRK1 and shut down SnRK1 signaling, thus activating TOR and promoting plant growth and development [80]. Moreover, TOR represses the activity of ABA receptors by phosphorylation, preventing SnRK2 activation and ABA downstream response [78]. During the induction of bud endodormancy in grapevine, ABA could induce *SnRK1* expression and inhibit cell respiration [81]. However, there is only limited evidence that SnRK1-TOR-SnRK2 cascade may be involved in bud dormancy in grapevine, further genetic evidence is still needed [82].

## 4. Cross-Talks between ABA and Other Hormones in Regulating Bud Dormancy

**Figure 3:**
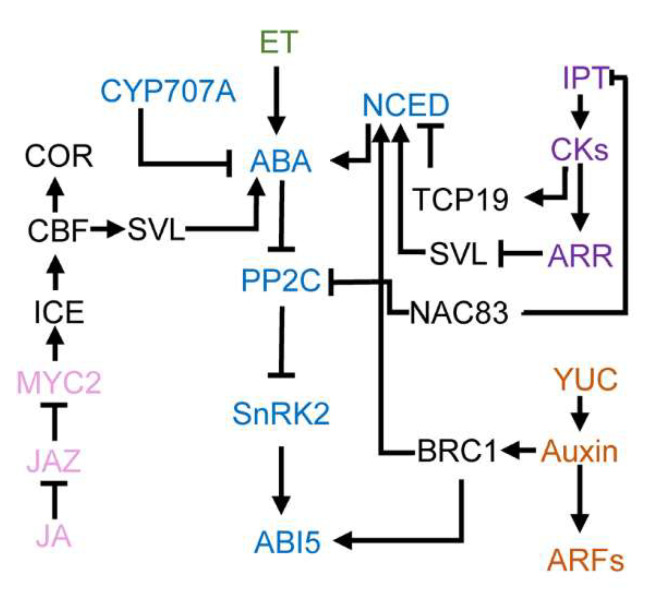
The crosstalks between ABA and other phytohormones in regulating bud dormancy release. Auxin and ABA crosstalk through BRC1-mediated hormone networks in axillary bud dormancy. BRC1 not only activates ABA signaling factors ABI5 but also up-regulated the expression of the key enzyme gene *NCED3* by combining with other proteins. CKs and ABA plays antagonistic role during bud dormancy release. Several transcriptional factors are involved in this process, such as TCP19, SVL and NAC83. In autumn, cold exposure activates JA biosynthesis, both JAZ and MYC2 interact with ICE, forming the ICE-CBF-COR cascade. As the CBF-DAM cascade regulates bud dormancy in popular, it is possible that the crosstalk between JA and ABA can be mediated by MYC2-ICE-CBF-DAM cascade, and induce/maintain bud dormancy. Ethylene has been shown to increase endogenous ABA and maintain bud dormancy in several species, but the mechanism is still need to be investigated. ABA metabolism and signaling is marked in blue; cytokinin metabolism and signaling is marked in purple; auxin metabolism and signaling is marked in orange; ethylene is marked in green; Jasmonic acid metabolism and signaling is marked in pink. Note that this overview summarizes interactions reported in different species or environmental condition, which are not necessarily occurring simultaneously. Full lines and dashed lines specify established.

Hormones, including gibberellin, cytokinins, ethylene, jasmonic acid, are also involved in bud dormancy in perennials, and there is cross-talk between ABA and these hormones in regulating perennial bud dormancy (Figure 3) [24,27,83,84].

### 4.1. ABA and Gibberellins

Gibberellins (GAs) stimulate cell division and elongation at different stages of plant development and in different tissues, including bud dormancy, seed germination, stem elongation, flowering and reproductive organs development [85,86,87]. There are more than 100 types of GAs, but only several forms are bioactive, e.g., GA_1_, GA_3_, GA_4_, and GA_7_ [85]. In GA metabolism, bioactive GAs are mainly catalyzed by GA 20-oxidases (GA20ox) and GA3ox. The existing bioactive GAs are deactivated to non-bioactive forms, which are catalyzed by GA2ox [88]. In GA signaling, the GA-GID1-DELLA module is considered to be universal in angiosperms. The binding of Gibberellins to the GA receptor GID1 (GIBBERELLIN-INSENSITIVE DWARF1) promotes interaction of GID1 with the DELLA proteins [24]. The GA-GID1-DELLA complex is recognized by the SCF^SLY1^/GID2 E3 ubiquitin-ligase, which triggers DELLA degradation by the ubiquitin-proteasome pathway [24]. DELLA proteins are classed into RGA (Repressor of Gibberellic Acid), GAI (Gibberellic Acid Insensitive) and RGA-like proteins by protein sequence similarities [89].

The effect of GA on bud dormancy is dependent on spatio-temporal and species’ variety [29,71,90]. In most cases, GA has a positive role in bud endodormancy release. In pear, exogenous GA could positively regulate bud endodormancy release that substitute for partial chilling treatment [91]. However, GA has a limited effect on *Gladiolus* corm dormancy release when treated at the early stage of endodormancy [92]. In Kiwi, application of GA_3_ before chilling promoted dormancy, while application after chilling promoted bud break [93]. GA is also involved in paradormancy regulation. The GA deficiency driven by increased expression of *GA2ox* resulted in increased axillary buds in hybrid aspen [94], whereas GA promotes the outgrowth of branches in *Jatropha curcas* [95].

Although the role of GA in the process of bud dormancy release and bud outgrowth needs to be further investigated, the antagonistic relationship between GA and ABA has long been shown to regulate key developmental processes, particularly seed dormancy and germination [96,97,98,99,100]. As for bud dormancy release, the antagonism between GA and ABA is also essential for integrating environmental and endogenous signals [44]. For example, in pears, *GAST1* (*GA-STIMULATED TRANSCRIPTS1*) integrates GA biosynthesis and ABA signaling and participates in pear bud dormancy release in winter [91]. GAST1 responds to GA and also plays positive role in GA biosynthesis [91,101]. Indeed, ABA can inhibit *GA20ox* by downregulation of *GAST1*, decreasing the level of GA and inhibiting bud dormancy release [91]. For shade avoidance in maize, ABA promotes while GA inhibits axillary bud dormancy (paradormancy) [102]. For bud endodormancy in popular inducted by SD and low temperature, SVL could act as the nexus of GA and ABA metabolism. SVL induces *GA2ox8* (a GA catabolic gene) and binds to the promoter of *NCED3* (an ABA biosynthesis gene), thereby inhibiting the growth of the buds [41,44]. A study in poplar showed that ectopically expressed RGL from Japanese Apricot delayed the onset of bud dormancy [83]. However, there is still lack of information about how GA signaling factors (e.g., RGL, RGA and GAI) crosstalk with or regulate ABA-related proteins in the process of perennial bud dormancy release.

### 4.2. ABA and Cytokinins

Cytokinins (CKs) are a group of adenine-derived compounds that are involved in stem and root meristem differentiation, seed germination, leaf senescence delay, stress response and bud dormancy [27,103,104,105]. Adenosine phospho-isopentenyl transferase (IPT) and CYP735As (CYTOCHROME P450, FAMILY 735, SUBFAMILY As) are key rate-limiting enzymes that catalyze CKs biosynthesis, and *tans*-Zeatin (tZ)-type cytokinins are one of the main types [106,107,108]. Cytokinin oxidase/dehydrogenase (CKX) is the main catalytic enzyme for CK degradation [109]. The core of CK signaling pathway is mainly composed of receptor histidine kinase (AHK), histidine phosphate transfer (HPt) proteins and type-A or type-B response (A-RR or B-RR) regulator, which initiate the expression of CK response genes [110,111,112,113].

In seed and corm dormancy, CKs antagonized ABA to promote seed germination, and a similar regulation has been found in buds [27,114]. First, CKs treatment broke the bud dormancy in strawberry axillary buds and tea buds, which was also shown in potato tubers, while GA_3_ was found to be insufficient to break dormancy when *CKX* was overexpressed (*CKX*-overexpressed tubers showed an extended dormancy period and did not respond to GA_3_), suggesting that CKs play an important role in terminating tuber dormancy and also indicating that GA is not sufficient to break dormancy in the absence of CK [115,116,117]. By detecting hormone levels before bud break, CKs was found to be the main regulator in the potato tuber buds in the switch from innate dormancy to the non-dormant state [118]. The willow bud dormancy is initiated by high content of ABA and low levels of CKs in the xylem sap [119]. Moreover, the CKs level reached the peak before bud-break, while the ABA content was decreased [120]. It was recently reported that tZ is at low level during bud dormancy and increased during flowering in pears, which supports a positive role for CKs in bud dormancy release [97].

The cross-talk between CKs and ABA in buds of woody plants is mainly through the *DAM* hub. Overexpression of *PmDAM6* increases the ABA content in the late dormancy and bud breaking stages, while decreasing CK level in *Prunus* [121]. CK-triggered responses down-regulate *MdoDAM1* via *MdoBRRs*, which further reduces ABA content and promotes the release of bud dormancy [122]. In addition to *DAM* hub, the enhancement of light-mediated CKs signals further negatively regulates ABA content in the dormant bud, indicating that light signal is also involved in the cross-talk between CKs and ABA [123,124,125,126]. It has also been reported that *AP2* inhibits meristem activity by negatively regulating CKs signaling, resulting in low mitotic activity and high expression of ABA-responsive genes [127]. Moreover, NAC83-PP2C module and TCP-NCED module indirectly affect the level of CKs by mediating ABA signaling and synthesis pathways, respectively, and jointly regulate corm dormancy in *Gladiolus* (Figure 3) [27,57].

### 4.3. ABA and Auxin

Auxin is mainly synthesized in shoot tips and young leaves and is involved in apical dominance/paradormancy, senescence, flowering and other developmental processes [128,129,130]. As one of the most abundant auxin, IAA synthesis starts from tryptophan, which is eventually oxidized to IAA by amino transferase and flavin monooxygenase (YUC), with YUC being an important rate-limiting enzyme in this process [130]. Auxin induces ARF-binding promoter expression by triggering Aux/IAA degradation [131].

Treatments with exogenous auxin, NAA, relieve the phloem dormancy by removing callose from the sieve tubes, resulting in bud dormancy release [132]. Low levels of free IAA were detected in tea buds at deep endodormancy stage, while high IAA levels were found during dormancy release, similar to what was found in Chinese fir, *Prunus mume* and grapevine buds [68,133,134,135]. In the underground vegetative buds of Canada thistle, auxin and ABA signals act as central regulators of developmental networks as well as paradormancy [136].

The formation of lateral branches can be divided into two steps: initiation of axillary meristems and outgrowth of axillary buds [137,138]. Auxin has long been considered a major signal of apical dominance, primarily inhibiting axillary bud growth [138,139]. The hormone network regulating axillary bud outgrowth mainly includes auxin, strigolactone (SL) and CKs [140,141,142]. Auxin indirectly inhibits the expression of *BRC1*, a promoter of axillary bud outgrowth, by inhibiting CKs biosynthesis genes while activating SL biosynthesis genes [143]. Auxin and ABA crosstalk through *BRC1*-mediated hormone networks in axillary bud dormancy (Figure 3). Exogenous ABA treatment inhibited branch development, while fluridone, an ABA biosynthesis inhibitor, promoted the development of branches of *Rosa hybrida* [142,144,145]. At the same time, plants with low ABA sensitivity produced more branches [146]. Therefore, ABA generally plays an inhibitory role in breaking dormancy in axillary buds [147].

ABA acts downstream of *BRC1* [147]. *BRC1* not only activates two ABA signaling factors *ABSCISIC ACID INSENSITIVE 5* (*ABI5*) and *ABF3* [148,149,150], but also up-regulates the expression of the key enzyme gene, *NCED3,* by interacting with other proteins [150,151]. In addition, endogenous auxin is inhibited in ABA-treated axillary buds, of which cell cycle-related genes including *CYCA2;1* and *PCNA1* (*POLIFERATING CELL NUCLEAR ANTIGEN1*) are repressed [147].

### 4.4. ABA and Ethylene

Ethylene (ET) is a simple gaseous hormone that regulates a wide range of plant developmental processes, including fruit ripening, seed germination, flowering, abscission, as well as bud dormancy [152,153]. ET biosynthesis starts from the amino acid methionine, which is first converted to SAM (S-adenoysl-methionine) by SAM synthase. SAM is then converted to 1-aminocyclopropane-1-carboxylic acid (ACC) by ACC synthase (ACS), and finally ET is produced by the conversion of ACC oxidase (ACO) [154,155,156,157]. In the absence of ET, the receptors (i.e., ETR1, ETR2, ERS1, ERS2 and EIN4) activate *Constitutive Triple Response 1* (*CTR1*) and inhibit ET signaling pathway by transmembrane protein EIN2 in Arabidopsis [156,158,159]. However, in the presence of ET, *CTR1* is inactivated, and the positive regulatory function of *ETHYLENE INSENSITIVE 2* (*EIN2*) is released [160,161]. EIN2 positively regulates the EIN3 transcription factor members in the nucleus [162,163]. The *ETHYLENE RESPONSE FACTOR* (*ERF*) genes, belonging to the AP2/ERF superfamily, are direct targets of *EIN3* and activate downstream ET responses [157,164,165].

Many studies have demonstrated that ET biosynthesis and signaling pathways regulate bud dormancy. ET signaling genes *EIN3*, *EIL1*, and *ERF* are abundant during the endodormancy and are decreased during the transition from endodormancy to ecodormancy in populus, suggesting that ET may play roles in dormancy maintenance and release [82,166]. The application of competitive ET antagonist 2,5-Norbornadiene (NBD) leads to premature sprouting in potato tubers [167]. In onion, exogenous ET not only affects the expression of *ACO* and receptors (*EIN4* and *EIL3*), but also increases the accumulation of ABA by up-regulating the expression of *NCED* [168]. The *etr1-4* mutant in birch had relative low ABA content and fast growth under SD. Moreover, *etr1-4* mutant is less sensitive to ABA when sprouting and has weaker paradormancy compared to wild-type plants, suggesting that ET signaling interacts with ABA signaling pathways in dormancy regulation [169]. In addition, ET interacts with GA to participate in bud dormancy by increasing DELLA accumulation in response to photochrome signals and upregulating *ERF6* expression [170,171]. However, exogenous ET was also reported to promote bud breaking in some species like grapevine, poplar, while NBD inhibited bud dormancy release and increased ABA level [56,172,173,174]. In pear buds, ET precursor ACC was gradually increased during bud break [175]. ERFs were significantly up-regulated in HC-treated buds or before bud-break [56,174], suggesting that ET may act antagonistically to ABA during bud dormancy release [24].

### 4.5. ABA and Jasmonic Acid

Jasmonic acid (JA) is a class of plant hormones that regulate plant development and defense processes [176]. The core part of its signaling pathway consists of JA ZIM-DOMAIN (JAZ) protein, F-box protein CORONATINE INSENSITIVE1 (COI1) and several groups of suppressed transcription factors (MYC2, MYC3, MYC4 etc.) [177,178,179]. JAs act synergistically with ABA by inhibiting DNA replication and activating the expression of anti-stress genes during stress tolerance, and exogenous JAs and ABA inhibit seed germination in several species [100,180,181,182,183].

JAs related genes were inhibited in bud dormancy and significantly upregulated in bud dormancy release, and the content of JAs was also significantly increased in bud dormancy release [184,185,186]. Whereas, during bud dormancy release in pear or lily bulbs, the JA content is gradually reduced with the decreases of ABA [175,187]. JA and ABA participate in the process of cold domestication together. In autumn, cold exposure activates JA biosynthesis, and both JAZ and MYC2 interact with ICE, forming the ICE-CBF-COR cascade discussed above. It is possible that the crosstalk between JA and ABA can be mediated by MYC2-ICE-CBF-DAM cascade, to induce/maintain bud dormancy (Figure 3) [188,189].

## 5. Epigenetic Regulation of Bud Dormancy Mediated by ABA

Epigenetics is the study of molecules and mechanisms that can perpetuate alternative gene activity states in the context of the same DNA sequence, including noncoding RNAs, DNA methylation, histone modification, heterochromatin, and 3D genome architecture [190]. As currently there is little information about heterochromatin and 3D genome architecture on bud dormancy, here we mainly summarize the knowledge of the former three types on perennial bud dormancy.

In plants, DNA methylation occurs in three different cytosine contexts: CpG, CHG and CHH, in which H can be either cytosine, thymine or adenine [191]. De novo methylation can occur by RNA-directed DNA methylation (RdDM), where small interference RNAs (siRNAs) guide DOMAINS REARRANGED METHYLTRANSFERASE 2 (DRM2) to homologous sequences in the genome [192]. Total levels of DNA methylation increased in dormant chestnut’s buds compared to non-dormant buds [193]. During bud dormancy release in sweet cherry, changes in DNA methylation precedes transcript changes, and responded to low temperatures including cold signaling, oxidation-reduction process, metabolism of phenylpropanoids, lipids and a *DAM* gene (i.e., *MADS1*) [194]. Long-term cold treatment stimulates DNA methylation in the promoter of *MADS1* in sweet cherry and increases siRNA that match this region, in accordance with the up-regulation of *FT* transcripts [191]. In Arabidopsis, miR402 could be induced by ABA and reduces the transcript levels of *DML3* (*DEMETER-LIKE PROTEIN 3*), which results in increased methylation levels at certain loci [195]. Currently, genes regulated by DNA methylation still need to be explored in the process of perennial bud dormancy.

Histone modification is a covalent post-translational modification that fine-tunes gene expression (activation or silencing) by altering chromatin structure, and includes methylation, acetylation, phosphorylation, ubiquitylation and sumoylation [196]. During the transition from dormancy to dormancy release in potato, muti-acetylation of H4 and H3.1/3.2 was increased [197]. Increased acetylation of H4 was also detected in the chestnut [193]. In peach, global H3K27me3 was higher in dormancy-released bud, compared with dormant buds. But, the modification is variable for specific gene [198]. Several studies showed histone modification is involved in bud dormancy by shifting on/off DAM/SVL in this duration [50,198,199]. Coinciding with cold accumulation in peach, several chromatin regions including the region around the translation start of *DAM6* in peach are marked by enriched H3K27me3, removal of H3K4me3 and acetylated H3 (H3ac) (Figure 1) [199]. Similar findings were found in Leafy spurge, kiwifruit and sweet cherry [200,201,202]. Other dormancy-related genes like *EARLY BUD BREAK3* (*EBB3*), a target of ABA signaling downstream of the SVL/ABA feedforward loop during dormancy release, is modified by H3K27me3 [55]. In addition, ABA promotes bud dormancy by inhibiting the expression of a chromodomain protein PICKLE (PKL), which inhibits SVL and promotes bud dormancy release in popular [33,41].

ncRNAs are functional RNAs that regulate gene expression at both the transcriptional and post-transcriptional levels, with many ncRNAs involved in histone modification, chromatin remodeling and DNA methylation [203]. In peach, several microRNAs, siRNAs and lncRNAs have been found to be correlated to bud dormancy release for instance miR319, miR6285, miR2275 and D4ncRNA (intronic ncRNA in *DAM4*) (Figure 1) [204]. Several long ncRNAs (>200 nt), isolated from popular buds at different dormant stage, were reported to be involved in regulating endodormancy release, for example, two lncRNAs acting as endogenous target mimics for gma-miRNA396h, which itself targets *CYP707As* [205].

## 6. Conclusions and Outlook

Dormancy is a complex phenomenon in plants and is also a ceasing trait. During bud dormancy, internal changes occur including deposition of plasmodesmatal callose, slow cell division, limiting import of sucrose, and changes of hormones. A large body of studies has shown that ABA is the hub that integrates environmental signals and endogenous chemicals to regulate bud dormancy in perennials (Figure 1). Recent research shows that SL and karrikin can be recognized by the receptor DWARF14 (D14) and KARRIKIN INSENSITIVE2 (KAI2), respectively, and these two receptors are homologs [206,207]. The fact that karrikin has been shown to be involved in bud and seed dormancy, as well as SL control axillary bud development, also suggests that these two hormones play a role in perennial bud dormancy [206,208]. It will be interesting to investigate the cross-talk between ABA and karrikin/SL in endodormancy of perennials. Moreover, it is also worthwhile to compare their role in paradormancy and endodormancy.

Although there are a few studies about epigenetics in regulating bud dormancy, several questions still need to be addressed. For example, how does endogenous ABA affect epigenetics in dormant buds during bud dormancy release with respect to heterochromatin and 3D genome architecture? How do ncRNA or histone modifications regulate ABA metabolism in perennial dormant bud?

When buds are in a dormant state, the plasmodesmata is blocked by callose. Is there any change among the organelles, like mitochondrial, amyloplast and golgi apparatus, during this biological process? How does ABA regulate the activity or development of these organelles?

It will be especially exciting to integrate genetics, omics (e.g., genomics, proteomics and metabolomics) and computational analyses to identify a broad and complex network of perennial bud dormancy, which helps elucidate the molecular components and mechanisms underlying multiple cellular and biological processes. By using omics techniques, it will be much easier to identify domestication loci associated with bud dormancy, which contributes to plant breeding via crossing or molecular methods.

## Data Availability

Not applicable.
